# Rapid production of pure recombinant actin isoforms in *Pichia pastoris*

**DOI:** 10.1242/jcs.213827

**Published:** 2018-04-23

**Authors:** Tomoyuki Hatano, Salvatore Alioto, Emanuele Roscioli, Saravanan Palani, Scott T. Clarke, Anton Kamnev, Juan Ramon Hernandez-Fernaud, Lavanya Sivashanmugam, Bernardo Chapa-y-Lazo, Alexandra M. E. Jones, Robert C. Robinson, Karuna Sampath, Masanori Mishima, Andrew D. McAinsh, Bruce L. Goode, Mohan K. Balasubramanian

**Affiliations:** 1 Centre for Mechanochemical Cell Biology and Division of Biomedical Sciences, Warwick Medical School, University of Warwick, Coventry CV4 7AL, UK; 2 Department of Biology, Brandeis University, Waltham, MA 02454, USA; 3 School of Life Sciences, University of Warwick, Coventry CV4 7AL, UK; 4 Institute of Molecular and Cell Biology, A*STAR (Agency for Science, Technology, and Research), Singapore 138673, Singapore; 5 Department of Biochemistry, National University of Singapore, Singapore 117597, Singapore; 6 Research Institute for Interdisciplinary Science, Okayama University, Okayama, 700-8530, Japan

**Keywords:** Actin, Actin purification, Biochemistry, Cytoskeleton

## Abstract

Actins are major eukaryotic cytoskeletal proteins, and they are involved in many important cell functions, including cell division, cell polarity, wound healing and muscle contraction. Despite obvious drawbacks, muscle actin, which is easily purified, is used extensively for biochemical studies of the non-muscle actin cytoskeleton. Here, we report a rapid and cost-effective method to purify heterologous actins expressed in the yeast *Pichia pastoris*. Actin is expressed as a fusion with the actin-binding protein thymosin β4 and purified by means of an affinity tag introduced in the fusion. Following cleavage of thymosin β4 and the affinity tag, highly purified functional full-length actin is liberated. We purify actins from *Saccharomyces* *cerevisiae* and *Schizosaccharomyces* *pombe*, and the β- and γ-isoforms of human actin. We also report a modification of the method that facilitates expression and purification of arginylated actin, a form of actin thought to regulate dendritic actin networks in mammalian cells. The methods we describe can be performed in all laboratories equipped for molecular biology, and should greatly facilitate biochemical and cell biological studies of the actin cytoskeleton.

## Abstract (precis)

**Summary:**Here, we describe a method to purify recombinant actin to homogeneity by expression in *Pichia pastoris*. The purified actin is polymerisation competent and should facilitate biochemical and cell biological studies of the actin cytoskeleton.

## INTRODUCTION

Actin is one of the most abundant eukaryotic proteins, and it assembles into one of the main cytoskeletal polymers in eukaryotic cells. The actin cytoskeleton is vast, and cells express hundreds of proteins that associate with actin to control its polymerization, stability and spatial organization, and harness it to a wide spectrum of biological tasks, including muscle contraction, intracellular transport, cell motility, cell and tissue morphogenesis, cell adhesion, transcriptional regulation and DNA damage (reviewed in Misu et al., 2017; Rottner et al., 2017; Skruber et al., 2018). As such, the study of actin has extended itself into almost all branches of biological research, creating a major need for biochemically characterizing the actin-regulatory effects of numerous proteins from diverse model organisms (e.g. yeast, flies, worms and mammals). To date, the majority of such studies have used skeletal muscle actin (isolated from rabbits or chickens), because large quantities can be isolated at a reasonable cost-effectiveness. However, there are some major drawbacks to using muscle actin, including the heterogeneity in its post-translational modifications, and key differences in the properties of muscle actin from non-muscle actin isoforms and actin in different species (Bergeron et al., 2010; Kijima et al., 2016; Müller et al., 2013; Takaine and Mabuchi, 2007; Ti and Pollard, 2011; Wen et al., 2008; Wen and Rubenstein, 2005). These considerations mean there is a need to develop new methods to purify actins, so that physiologically relevant biochemical and cell biological studies may be undertaken.

In this paper, we describe a method to purify recombinant actin through expression in the yeast *Pichia pastoris*. We purify *Saccharomyces* *cerevisiae*, *Schizosaccharomyces* *pombe*, and β- and γ-isoforms of human actin in a form that is functional *in vitro* and *in vivo*.

## RESULTS

**Fig. 1.**
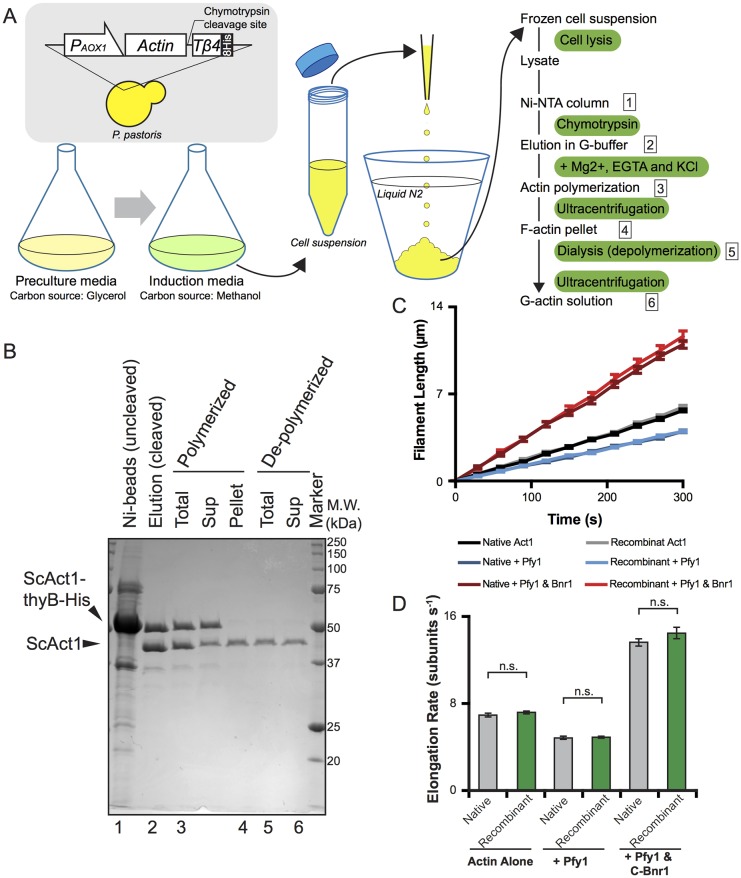
**Purification of assembly-competent recombinant *Saccharomyces cerevisiae* Act1 from *Pichia pastoris*.** (A) Diagram of the actin purification method (for details see Materials and Methods). (B) Coomassie-stained gel (CBB) showing purified recombinant *Saccharomyces cerevisiae* Act1. Numbering (1–6) at the bottom corresponds to the steps designated by numbers in the flow chart in A. (C) TIRF microscopy analysis of actin filament growth, comparing Act1 purified from *S. cerevisiae* (native) and recombinant Act1 (recombinant) purified from *P. pastoris*, in the presence or absence of *S. cerevisiae* profilin (Pfy1) and formin (Bnr1). All reactions contained 10% Oregon Green-labelled skeletal muscle actin as a tracer. Data are averaged from three independent experiments (*n*=10 filaments per experiment; 30 total). Error bars show the s.e.m. (D) Actin filament elongation rates calculated from reactions in C. n.s., not significant (Student's *t*-test).

To develop an easy and cost-effective method for purification of recombinant actins, we used the methylotrophic yeast *P. pastoris*, which is an excellent organism to use for the expression of heterologous proteins (Gellissen, 2000; Macauley-Patrick et al., 2005). To reduce cross-contamination with endogenous *P. pastoris* actin, we introduced a strategy that blocks co-polymerization of recombinant actin with the host actin both during cell growth and during purification. This was achieved by fusing the actin monomer-binding protein thymosin β4 (Tβ4; also known as TMSB4X) sequence to the C-terminus of the actin-coding sequence, with a short intervening in-frame linker (schematic in Fig. 1A; Noguchi et al., 2007). Fusion of actin to Tβ4 leads to intramolecular interactions preventing polymerization of the heterologous actin. A His tag (eight consecutive histidine residues, 8His) was further added to the C-terminus of the actin–Tβ4 fusion to facilitate affinity purification. Importantly, since the actin-coding sequence ends with a highly conserved phenylalanine residue (a cleavage site for chymotrypsin), and since all other phenylalanine residues are buried (Jacobson and Rosenbusch, 1976), incubation of the fusion protein with chymotrypsin releases native (untagged) actin. This Tβ4 fusion purification approach has been used for heterologous actin expression in *Dictyostelium* and insect cells (Huang et al., 2016; Kijima et al., 2016; Noguchi et al., 2007; Xue et al., 2014). However, we reasoned that heterologous actin expression in *P. pastoris* would provide a much easier alternative that could be used in virtually any laboratory equipped for molecular biology.

As a test case, we first expressed actin from *S. cerevisiae* (UniProtKB entry: P60010, ACT_YEAST) as the actin cytoskeleton from this organism has been extensively characterized through genetics, biochemistry and microscopy (Engqvist-Goldstein and Drubin, 2003; Moseley and Goode, 2006). Furthermore, since several biochemical studies in *S. cerevisiae* have used native actin purified from yeast cells, we reasoned that the vast existing knowledge of *S. cerevisiae* actin properties would help in the functional evaluation of the heterologously expressed *S. cerevisiae* actin. We were able to purify *S. cerevisiae* actin to near homogeneity after only a small number of steps, including binding to Ni-NTA resin, cleavage with chymotrypsin, and a cycle of polymerization and depolymerization (Fig. 1B). The Ni-NTA-bound fraction did not contain a prominent band at ∼42 kDa, suggesting that native *P. pastoris* actin (UniProtKB entry: Q9P4D1, ACT_KOMPG) did not appreciably co-purify in this process (Fig. 1B). Next, we compared the polymerization properties of *S. cerevisiae* actin purified from *S. cerevisiae* (‘Native’) versus *S. cerevisiae* actin purified from *P. pastoris* (‘Recombinant’). We found that actin filament elongation rates were nearly identical, both in the presence and absence of a yeast formin (Bnr1) and yeast profilin (Pfy1) (Fig. 1C,D). As expected, addition of Pfy1 alone, without the formin, marginally reduced the rate of elongation of native and recombinant Act1 polymers (Fig. 1C,D), which we have also observed for profilins from several different species (Chesarone and Goode, 2009; Chhabra and Higgs, 2007). We also found that there was no difference in average filament length formed over time between the native and recombinant Act1 (Fig. 1C,D). Based on these results, we conclude that recombinant Act1 has polymerization properties that are comparable to those of native Act1, and thus can be used reliably for biochemical studies.

**Fig. 2.**
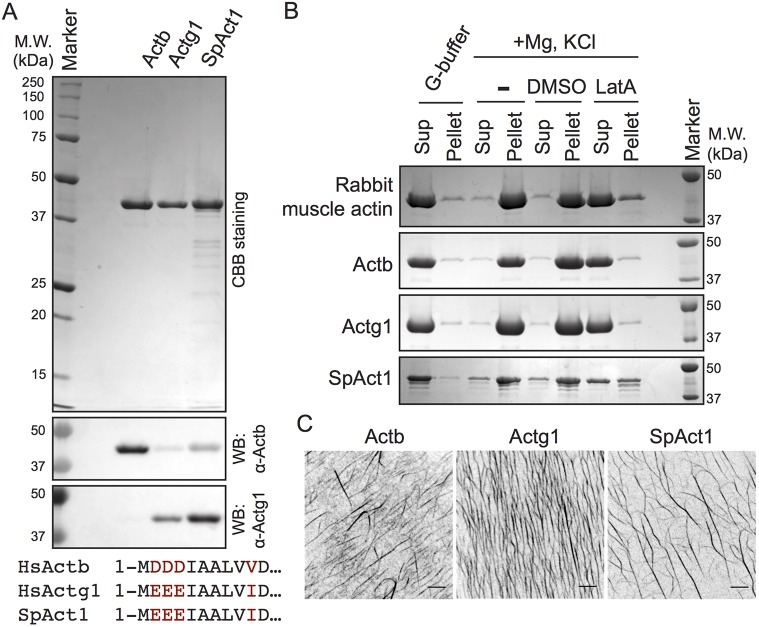
**Purification of recombinant human non-muscle actins and fission yeast Act1.** (A) CBB gel image showing purified recombinant human β-actin (Actb), human γ-actin (Actg1) and *Schizosaccharomyces pombe* (SpAct1) actin. Immunoblotting in the bottom panels was performed with antibodies against Actb or Actg1 raised against peptides corresponding to the N-terminus of human β- and γ-actins. Since the N-terminus of *S. pombe* Act1 has strong similarity to human γ-actin, the anti-Actg1 antibody can recognize *S. pombe* Act1 as well as γ-actin (see sequence alignment shown at the bottom). (B) Actin sedimentation assays. Purified actin in low-ionic strength buffer (G-buffer) was mixed with KCl, EGTA and Mg^2+^ to induce actin polymerization. Actin sedimentation was tested in the presence of 0.5 mM LatA or in solvent control (DMSO) and in the absence of DMSO or Latrunculin A (‘−’). After ultracentrifugation, actin in the pellet (Pellet) and supernatant (Sup) fractions were detected by CBB staining following SDS-PAGE. Rabbit muscle actin was used as a positive control. (C) Visualization of F-actin. The specified actins were incubated with KCl, EGTA and Mg^2+^ in the presence of 2% PEG. F-actin in all cases was then stained with Rhodamine–phalloidin and imaged by using a Nikon inverted microscope fitted with a Yokogawa spinning-disc head and an Andor iXon camera. Scale bars: 5 µm.

To test the versatility of the heterologous actin expression system, we also attempted to express and purify fission yeast *S. pombe* actin (UniProtKB entry: P10989, ACT_SCHPO, hereafter denoted SpAct1), since this organism has been used extensively in studies of the actin cytoskeleton and cytokinesis (Mishra et al., 2014; Pollard and Wu, 2010). We expressed and purified SpAct1 as an actin–Tβ4–His fusion using the same steps as used to purify *S. cerevisiae* actin (Fig. 2A). Similar to what was seen for *S. cerevisiae* Act1, the Ni-NTA-bound fraction did not show major contamination with *P. pastoris* actin (Fig. S1A, lane marked as uncleaved). N-terminal integrity was confirmed by immunoblotting with human anti-γ-actin antibodies; *S. pombe* actin was recognized by antibodies against human γ-actin, consistent with the N-terminal sequence conservation between *S. pombe* and human γ-actin (Fig. 2A). We note that SpAct1 was more prone to proteolysis than *S. cerevisiae* Act1 (Fig. 2A), possibly reflecting some exposure of additional chymotrypsin sites.

We next asked whether we could express and purify the two human non-muscle actin isoforms, β- and γ-actin (UniProtKB entries: P60709, ACTB_HUMAN and P63261, ACTG_HUMAN, respectively). It is noteworthy that preparations of human non-muscle actins expressed in insect cells typically contain co-purifying host actin (Bergeron et al., 2010), and actin purified from human non-muscle cells or animal sources contains a mixture of β- and γ-isoforms. Similar to what was seen for the yeast actins, the Ni-NTA-bound fraction showed no major contamination with *P. pastoris* actin (Fig. S1A, lane marked as uncleaved). Mass spectrometric analysis of β- and γ-actin detected all peptides derived from trypsin or chymotrypsin digestion of the expressed actins including unique N-terminal peptides. Mass spectrometry (MS) results are shown in Tables S1 and S2, and homogeneity of the purified actin is discussed in the legend to Fig. S2 (see also Fig. 4C). Immunoblotting of the purified actins with monoclonal antibodies against human β- and γ-actin supports N-terminal integrity of these proteins (Fig. 2A).

To test the biochemical activities of purified SpAct1, and the human β- and γ-actins, we performed sedimentation assays in the presence or absence of the actin polymerization inhibitor Latrunculin A (LatA) (Fig. 2B). In low-ionic strength buffer (G-buffer), as was observed with rabbit muscle actin (Fig. 2B, top panel), all recombinant actins remained in the supernatant fraction after ultracentrifugation. However, under polymerization conditions, after the addition of MgCl_2_ and KCl, the muscle actin and recombinant actins shifted primarily to the pellet fractions indicating polymerization (Fig. 2B). Furthermore, although DMSO solvent alone did not affect pelleting, incubation with LatA blocked the polymerization of all actins. Furthermore, *in vitro* polymerization assays, as monitored by Rhodamine–phalloidin staining, demonstrated that the recombinant *S. pombe* and human β- and γ-actins polymerized normally into filaments (Fig. 2C).

**Fig. 3.**
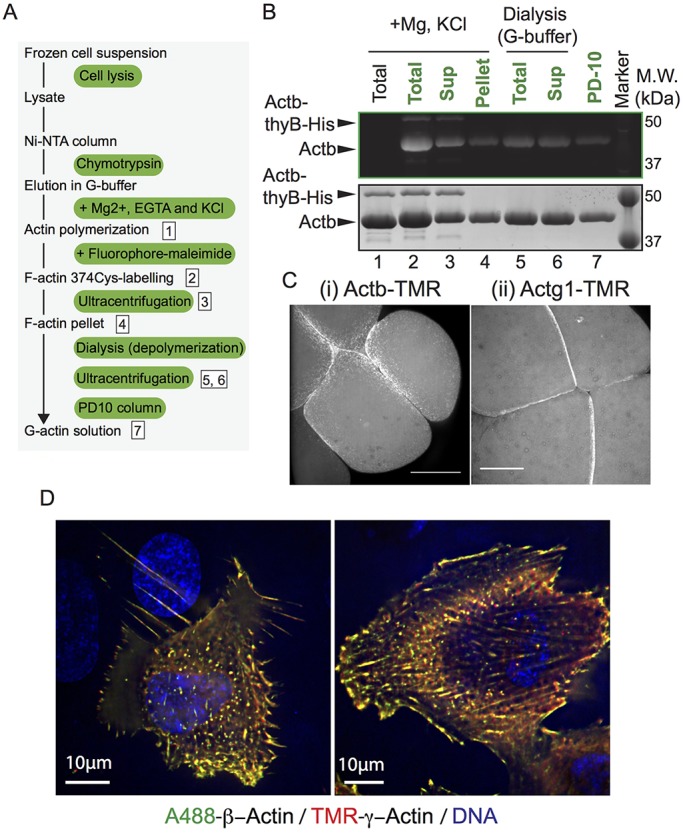
**Recombinant actins were incorporated into actin cytoskeleton networks in cells.** (A,B) Schematic representation and gel image of labelling method for actin Cys^374^ (details are shown in Materials and Methods). Eluates collected after chymotrypsin treatment (see Fig. 1A) were mixed with KCl and Mg^2+^ to induce polymerization of β-actin (lane 1) and incubated with Alexa Fluor 488 C5 maleimide to label the residue 374 of β-actin in F-actin (lane 2). The reaction mixture was spun in an ultracentrifuge to pellet F-actin [lane 3, supernatant (Sup); lane 4, pellet fraction (Pellet)]. The pellet fraction was suspended in low-salt G-buffer, and dialyzed against the same G-buffer. The dialyzed sample (lane 5) was ultracentrifuged to remove F-actin (lane 6). Free Alexa Fluor 488 C5 maleimide was removed by means of a desalting column (lane 7, G-25). (C) Microinjection of β-actin (i) and γ-actin (ii) labelled with TMR into zebrafish embryos. Images were acquired using a spinning-disc confocal microscope. Scale bars: 100 µm. (D) Injection of fluorescently labelled β- and γ-actins into RPE1 cells. β- and γ-actin were labelled with Alexa Fluor 488 and TMR, respectively and injected into human RPE1 cells. The left panel shows a maximum projection of all *z*-axis stacks. The right panel shows a maximum projection of *z*-axis stacks 24–26.

To examine whether β- and γ-actins can be incorporated into cellular actin cytoskeletal networks, we labelled β- and γ-actins with Alexa Fluor 488 and Tetramethylrhodamine (TMR), respectively (schematic representation of the strategy is shown in Fig. 3A and β-actin labelled with Alexa Fluor 488 is shown in Fig. 3B), and injected into zebrafish embryos. We found that both actin isoforms could be efficiently incorporated into the actin cytoskeleton at cell–cell contacts (Fig. 3C). In addition, we injected β- and γ-actins labelled with Alexa Fluor 488 and TMR, respectively, into human RPE1 cells. The injected actins colocalized and assembled into native actin cytoskeleton-like structures, and time-lapse imaging revealed their incorporation into dynamic filamentous structures (Fig. 3D; Movie 1). From these results, we conclude that the labelled β- and γ-actins are active and readily decorate filamentous actin networks in RPE cells and zebrafish embryos.

**Fig. 4.**
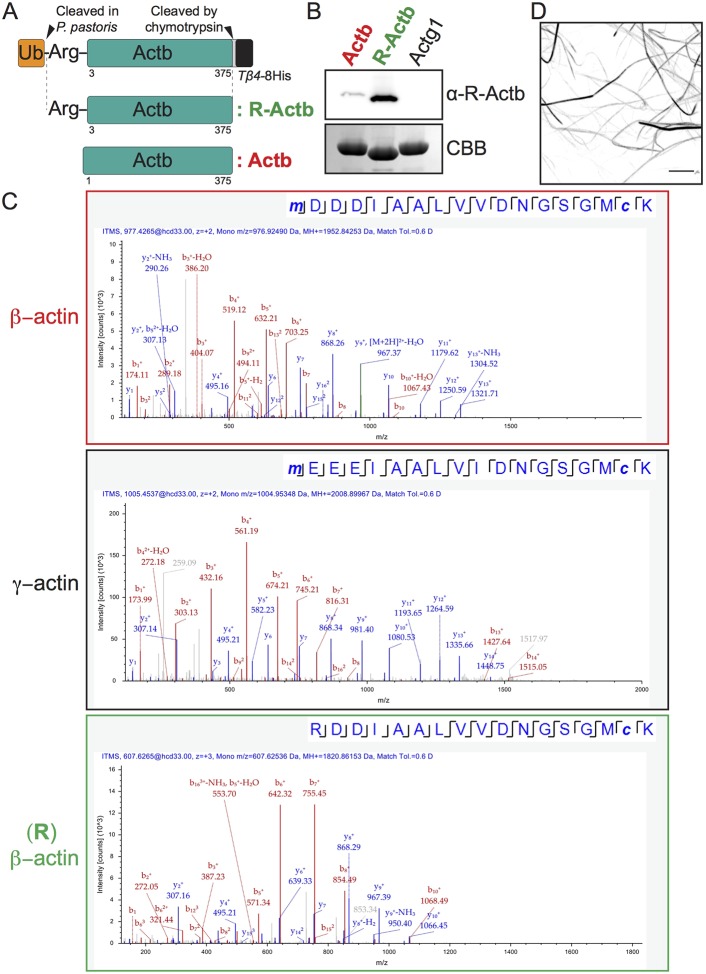
**Purification of arginylated β-actin.** (A) Schematic representation of expression of arginylated β-actin (R-Actb). (B) Immunoblotting and CBB staining showing purified R-Actb. Immunoblotting was performed with polyclonal antibodies against arginylated β-actin. (C) Representative mass spectra of the unique N-terminal tryptic peptide of β-actin, γ-actin and arginylated β-actin [(R) β-actin] as obtained after HCD fragmentation. The N-terminal (R) β-actin tryptic unique peptide shows the replacement of Met-Asp by Arg. (D) Visualization of arginylated actin (R-Actb) filament. R-Actb was incubated with KCl, EGTA and Mg^2+^ in the presence of 2% PEG and stained with Cy5–phalloidin. Scale bar: 5 µm.

Having established a simple method to purify large quantities of heterologous actins in *P. pastoris*, we next adapted this method to the production of arginylated actin, since human β-actin exists in both arginylated and non-arginylated forms (Karakozova et al., 2006). Arginylated actin appears to play key roles in actin filament assembly in the leading edge dendritic networks. To express and purify arginylated β-actin (denoted R-β-actin), we created a construct that directs expression of a quadruple fusion containing *S. cerevisiae* ubiquitin and human R-β-actin, as well as Tβ4 and His (ubiquitin–R-β-actin–Tβ4–His) (Fig. 4A). This fusion protein was expressed in *P. pastoris* and the ubiquitin moiety was cleaved by the endogenous ubiquitin-specific protease (Bachmair et al., 1986). Human R-β-actin–Tβ4–His was then purified as before and following cleavage with chymotrypsin, and was isolated to near homogeneity (results of mass spectrometric analysis are shown in Tables S1 and S2 and the assessment of the homogeneity of the purified actin is discussed in the legend to Fig. S2). An antibody against arginylated actin strongly recognized R-β-actin but only very weakly recognized unmodified β-actin (Fig. 4B). Mass spectrometric analysis detected the N-terminal arginylated peptide (Fig. 4C; Table S1). These results strongly indicate the N-terminal integrity of R-β-actin. Note that R-β-actin runs slightly faster on SDS-PAGE gels, probably reflecting different structural states of unmodified and arginylated β-actin in complex with SDS (Fig. 4B; Fig. S1B). The faster migration of R-β-actin has also been reported by other investigators (Saha et al., 2010). We also found the R-β-actin could be polymerized *in vitro*, suggesting that R-β-actin purified from *P. pastoris* is functional (Fig. 4D).

## DISCUSSION

In summary, we have developed a simple and rapid method to purify large quantities of any specific actin isoform that requires only standard laboratory equipment. The plasmids we describe will be deposited in Addgene. Investigators should be able to clone any actin gene of interest into these vectors, transform *P. pastoris*, and quickly purify the actin for biochemical characterization, and/or labelling and introduction into cells for live imaging.

We successfully purified human non-muscle β- and γ-actins, and actins from *S. cerevisiae* and *S. pombe*. Approximately 0.5–1 mg of actin is purified from a 1 l culture of cells grown in standard flasks (∼10 g of cells from 1 l saturated culture). By using the method we describe, actin from virtually any eukaryotic organism can be isolated and used to study the biochemical effects of actin-binding proteins from the same species, improving the physiological relevance of the results. This method will also enable studies comparing the different actin isoforms, isolated to homogeneity, in the absence of other contaminating isoforms. Furthermore, we used this method to produce arginylated actin, which opens up exciting avenues for characterization of actins with different post-translational modifications (Karakozova et al., 2006; Saha et al., 2010; Zhang et al., 2010). Finally, this method will enable purification to homogeneity of actins carrying point mutations that cause different human diseases (Laing et al., 2009; Nowak et al., 1999; Rivière et al., 2012), and the subsequent use of these purified mutants in biochemical drug screens. Thus, basic and translational studies will be positively impacted by the simple actin purification method we describe.

Actins undergo a variety of post-translational modifications and although some post-translational modifications occur in *P. pastoris*, other modifications do not. In future, it will be important to develop synthetic biology tools (such as expression of mammalian actin acetyl transferases and methyl transferases as well as genetic code expansion for incorporation of modified amino acids) to express and purify actins carrying other modifications. Such tools will both facilitate the elucidation of function of various actin modifications as well as promote physiologically relevant biochemical and cell biological experiments.

## MATERIALS AND METHODS

### Plasmids used in this study

Actin-coding sequences were cloned into pPICZc (Invitrogen), which adds, at the C-terminus, an in-frame chymotrypsin cleavage site, linker sequence, thymosin β4 and a His tag (Noguchi et al., 2007). These included *Homo sapiens* ACTB cDNA, synthesized *H. sapiens* ACTG1 cDNA (codon optimized for *P. pastoris*), the *S. pombe act1* gene, and the *S. cerevisiae ACT1* gene (codon optimized for *P. pastoris*).

### SDS-PAGE and immunoblotting

Samples were fractionated on 12% SDS-PAGE gels (acrylamide:bis-acrylamide 37.5:1, #1610158, Bio-Rad), and stained with Coomassie Brilliant Blue (CBB) via InstantBlue (Expedeon). For immunoblotting, proteins were transferred onto nitrocellulose membranes using a semi-dry blotting system (Trans Blot Turbo, Bio-Rad). Membranes were incubated at room temperature for 1 h in blocking buffer (5% skimmed milk in TBS containing 0.5% Tween 20 and 0.02% NaN_3_), then incubated in 0.001% (v/v) anti-Actb (#MABT825, Merck Millipore) or Actg1 (#MABT824, Merck Millipore) antibodies in blocking buffer at 4°C for 2 h. Membranes were washed four times with TBS containing 0.5% Tween 20, then incubated with 0.003% (v/v) anti-mouse-IgG antibody conjugated to horseradish peroxidase (HRP). For the immunoblotting in Fig. S2B, HRP-conjugated antibodies against human actin C-terminus (1:1000, #sc-1616, Santa Cruz Biotechnology) was used to detect β-, γ- and arginylated β-actin. Proteins were detected with the ECL western blotting detecting reagent (#RPN2106, GE Healthcare).

### *P. pastoris* culture and transformation

The composition of the MGY and MM growth media and basic techniques for *P. pastoris* X-33 are described in the Pichia Expression Kit Instruction Manual (https://www.thermofisher.com/order/catalog/product/V19020). *P. pastoris* X-33 was transformed with pPICZc-ACTB, ACTG1 or *S. pombe act1* or *S. cerevisiae ACT1*. Plasmid DNA was linearized with PmeI and transformed by using the lithium chloride method. Transformants were selected on yeast extract peptone dextrose (YPD) plates containing 100 mg/l Zeocin (Gibco, #R25001) grown at 30°C.

### Purification of recombinant actins from *P. pastoris*

*P. pastoris* transformants were revived on YPD solid medium. Cells were inoculated into 200 ml minimal glycerol (MGY) liquid medium composed of 1.34% yeast nitrogen base without amino acids (Sigma, Y0626), 0.00004% biotin and 1% glycerol and cultured at 30°C, shaking at 220 rpm. Culture medium was diluted 1.5 l by addition of 1.3 l fresh MGY medium, and cells were cultured at 30°C, with rotation at 220 rpm, until the optical density at 600 nm (OD_600_) reached 1.5. Cells were pelleted by centrifugation at 10628 ***g*** at 25°C for 10 min (Thermo Fisher Scientific, #F9-6×1000 LEX rotor). Cells were washed once with sterilized water and re-suspended into 2 l minimal methanol (MM) medium, composed of 1.34% yeast nitrogen base without amino acids (Sigma, Y0626), 0.00004% biotin and 0.5% methanol. Cells were cultured in four separate 2 l flasks (500 ml culture each) at 30°C, with rotation at 220 rpm, for 1.5–2 days; 0.5% methanol was added every 24 h of culturing. Cells were pelleted as above, yielding ∼10 g wet weight of cells per 1 l culture. Cells were washed once with water and resuspended in 30 ml water. The suspension was dripped into liquid nitrogen to form frozen beads, which were stored at −80°C until use.

For each preparation, 30 g of frozen cells was used, which was loaded into eight grinder tubes (#6751, SPEX^®^ SamplePrep) pre-cooled with liquid nitrogen and ground in a Freezer mill (#6870, SPEX^®^ SamplePrep) in a liquid nitrogen bath. The duration of the grinding was 1 min with 14 cycles/second. The grinding was repeated 30 times at 1 min intervals. Liquid nitrogen was re-filled every ten cycles of grinding. The lysate powder was resolved in an equal amount of 2× binding buffer [20 mM imidazole, 20 mM HEPES pH 7.4, 600 mM NaCl, 4 mM MgCl_2_, 2 mM ATP, 2× concentration of protease inhibitor cocktail (cOmplete, EDTA free #05056489001, Roche), 1 mM phenylmethylsulfonyl fluoride (PMSF) and 7 mM β-mercaptoethanol (β-ME)]. The lysate was sonicated on ice (10 s with 60% amplitude, Qsonica Sonicators) until all aggregates were resolved. The lysate was centrifuged at 4°C at 3220 ***g*** for 15 min (Eppendorf #A-4-81 rotor) to remove intact cells and debris, then further cleared by centrifugation at 4°C at 25658 ***g*** for 1 h (Thermo Fisher Scientific, #A23-6×100 rotor). The supernatant was passed through a 0.22 µm filter (Corning #431097) and incubated with 1 ml nickel resin (Thermo Scientific, #88222) at 4°C for 1–1.5 h. The resin was pelleted down by centrifugation at 4°C at 1258 ***g*** for 5 min (Eppendorf #A-4-81 rotor) and washed with 25 ml ice-cold binding buffer composed of 10 mM imidazole, 10 mM HEPES pH 7.4, 300 mM NaCl, 2 mM MgCl_2_, 1 mM ATP, protease inhibitor cocktail (cOmplete, EDTA free #05056489001, Roche), 1 mM PMSF and 7 mM β-ME. The resin was loaded into an open column (Bio-Rad, #731-1550) and washed first with ice-cold 20 ml binding buffer, then with 45 ml ice-cold G-buffer composed of 5 mM HEPES (pH 7.4), 0.2 mM CaCl_2_, 0.01% (w/v) NaN_3_, 0.2 mM ATP and 0.5 mM dithiothreitol (DTT). The resin was resuspended in 6 ml ice-cold G-buffer containing 5 µg/ml TLCK-treated chymotrypsin (Sigma, #C3142-10MG) and incubated overnight at 4°C. The chymotrypsin was inactivated by addition of PMSF to 1 mM and incubated for 30 min on ice. The eluate was then collected into a tube. Actin retained on the resin was eluted with 12 ml G-buffer and all the elution fractions were combined. The eluate was concentrated using a 30 kDa cut-off membrane (Sigma-Aldrich, #Z677892-24EA) and the final volume adjusted to 900 µl with ice-cold G-buffer. Actin was polymerized by addition of 100 µl 10× MEK buffer, composed of 20 mM MgCl_2_, 50 mM glycol-bis(2-aminoethylether)-N,N,N′,N′-tetraacetic (EGTA) and 1 M KCl, for 1 h at room temperature. F-actin was pelleted by ultracentrifugation for 1 h at room temperature at 45,000 rpm (Beckman TLA-55 rotor) and re-suspended in 1 ml ice cold G-buffer. F-actin was depolymerized by dialysis against 1 l G-buffer at 4°C for 2 days. Dialysis buffer was exchanged every 12 h. Any remaining F-actin was pelleted by ultracentrifugation at room temperature at 45,000 rpm for 30 min (Beckman TLA-55 rotor) and actin in the supernatant was concentrated to 100 µM using a 30 kDa cut-off membrane (Sigma-Aldrich, #Z677892-24EA). The concentration of actin was determined by measuring the absorbance at 290 nm [*A*290=0.63 (/mg/ml/cm)] using a NanoDrop 2000c spectrophotometer (Thermo Fisher Scientific).

### Purification of native yeast Act1 and recombinant Pfy1 and Bnr1

Act1 was isolated from *S. cerevisiae* as described previously (Cook et al., 1992), except using G-buffer consisting of 5 mM HEPES (pH 7.6), 0.2 mM CaCl_2_, 0.2 mM ATP, 2 mM β-mercaptoethanol. Rabbit muscle actin was purified from acetone powder following a protocol from Pardee and Spudich (1982). Rabbit muscle actin was labelled with Oregon Green (OG), and the concentration and labelling efficiency were determined as described (Kuhn and Pollard, 2005). 6His-tagged Bnr1 (FH1-FH2-C) was overexpressed in *S. cerevisiae* and purified by nickel chromatography and gel filtration as described (Moseley et al., 2006). Untagged *S. cerevisiae* profilin (Pfy1) was expressed and purified from *E. coli* by anion-exchange chromatography and gel filtration as described (Moseley et al., 2004).

### TIRF microscopy

Glass coverslips (24×60 mm #1.5; Fisher Scientific, Pittsburgh, PA) were sonicated for 1 h in 2% Micro-90 detergent, followed by 1 h sonication in 100% ethanol, then 30 min sonication in 0.1 M KOH and double-distilled (dd)H_2_O, respectively. Cleaned coverglass was stored in 100% ethanol prior to use. Before each experiment, coverslips were rinsed with ddH_2_O, dried with N_2_, and coated by applying 120 µl of 2 mg/ml methoxy-poly(ethylene glycol) (mPEG)-silane MW 2000 (Laysan Bio, Arab, AL) and 4 µg/ml biotin-PEG-silane MW 3400 (Laysan Bio, Arab, AL) resuspended in 80% ethanol, pH 2.0. Coated coverslips were incubated for 16 h at 70°C. Flow cells were assembled by rinsing PEG-coated coverslips with ddH_2_O, drying with N_2_, and adhering to µ-Slide VI0.1 (0.1 mm×17 mm×1 mm) flow chambers (Ibidi, Martinsried, Germany) with double-sided tape (2.5 cm×2 mm×120 µm) sealed with 5-min epoxy resin (Devcon, Riviera Beach, FL). Flow cells were incubated for 1 min with 1% BSA, then incubated for 1 min with total internal reflection fluorescence (TIRF) buffer [10 mM imidazole pH 7.4, 50 mM KCl, 1 mM MgCl_2_, 1 mM EGTA, 0.2 mM ATP, 10 mM DTT, 15 mM glucose, and 0.25% methyl cellulose (4000 cP)]. TIRF reactions were initiated by adding 1 µM actin (20% OG-labelled) to premixed actin-binding proteins. Reactions were introduced into the flow chamber, which was then mounted on the microscope stage for imaging. The time between addition of actin and the start of TIRF recording was 180 s. Time-lapse imaging was performed on a Nikon-Ti200 inverted microscope (Nikon Instruments) equipped with a 488 nm argon laser (150 mW; Melles Griot, Carlsbad, CA), a 60× Apo oil-immersion TIRF objective (NA 1.49; Nikon Instruments), and an EMCCD camera with a pixel size of 0.267 µm (Andor), and running NIS-Elements (Nikon Instruments). Focus was maintained by means of the Perfect Focus system (Nikon Instruments). Images were collected at 5 s intervals for 15 min. Background fluorescence was removed from each image in a stack by using the background subtraction tool (rolling ball radius, 50 pixels) in ImageJ. Minimal contrast enhancement or changes to the black level were applied to the entire stack to improve image quality for analysis and display. Filament elongation rates were determined from *n*≥10 filaments in each of three independent experiments. Filament length was measured by using the freehand line tool in ImageJ. Elongation rates were determined by plotting filament length versus time, where the rate is the slope. To express rates in actin subunits s^−1^, we used the conversion factor of 374 subunits per micrometre of F-actin.

### Cysteine labelling with fluorophore-conjugated maleimide

For experiments in which we introduced Alexa-Fluor-488–actin or TMR–actin into cells, we directly labelled Cys^374^, the C-terminal residue of actin. Actin released by chymotrypsin digestion and eluted from the nickel resin (see purification details above) was concentrated to ∼1.3 mg/ml, and then polymerized for 1 h at 4°C by addition of 2 mM MgCl_2_ and 100 mM KCl. Alexa Fluor 488-C5-conjugated maleimide (Thermo Fisher Scientific, #A10254) or Tetramethylrhodamine (TMR)-5-conjugated maleimide (Thermo Fisher Scientific, #T6027) dissolved in DMSO as a 3 mM stock was added drop-wise to reach a 3.5 molar excess of dye to actin, and incubated for 1 h at room temperature in the dark. The reaction was quenched by addition of 10 mM DTT, and the reaction was cleared of any precipitated dye by centrifugation at 4°C at 5000 rpm for 10 min (Eppendorf FA-45-24-11). F-actin was pelleted by ultracentrifugation at room temperature at 45,000 rpm for 1 h (Beckman TLA-55 rotor). The pellet was re-suspended and dialyzed against G-buffer to depolymerize actin, as described above, and free fluorophore-conjugated maleimide was separated from labelled actin by using a Sephadex G-25 (PD MidiTrap G-25, GE Healthcare #28-9180-07) column. The concentration of fluorophore-labelled actin was measured by determining the absorbance at 290 nm (actin), 495 nm (Alexa Fluor 488) and 550 nm (TMR) on a NanoDrop 2000c spectrophotometer (Thermo Fisher Scientific), and calculated using the following formula: actin concentration (M)=*A*290/(0.63×42,000). The extinction coefficient of Alexa Fluor 488 at 495 nm is *A*495=72,000 M^−1^ cm^−1^, with a correction factor for Alexa Fluor 488 absorbance at 290 nm of 0.138. Labelling efficiency was calculated as follows: (*A*495/72,000)/actin concentration (M). Purified actin was kept on ice in the dark.

### MS methods and analysis

50 µg of each purified protein were separated on a 12% SDS-PAGE gel and stained with Coomassie G-250 (SimplyBlue SafeStain, Invitrogen). The actin gel band (∼42 kDa) was cut and in-gel digested with trypsin and peptides concentrated and desalted on StageTips (1) reversed phase chromatography with two columns (Rappsilber et al., 2003), an Acclaim PepMap µ-precolumn cartridge of 300 µm internal diameter (i.d.) × 5 mm length, 5 μm particle diameter and 100 Å pore size, and an Acclaim PepMap RSLC of 75 µm i.d. × 50 cm length, 2 µm particle diameter and 100 Å pore size (Thermo Scientific), which were used to separate tryptic peptides prior to mass spectrometric analysis. The columns were installed on an Ultimate 3000 RSLCnano system (Dionex) at 40°C. Mobile phase buffer A was composed of 0.1% formic acid and mobile phase B was composed of acetonitrile containing 0.1% formic acid. Samples were loaded onto the µ-precolumn equilibrated in 2% aqueous acetonitrile containing 0.1% trifluoroacetic acid for 5 min at 10 µl min^−1^, after which peptides were eluted onto the analytical column at 250 nl min^−1^ by increasing the mobile phase B concentration from 6% B to 25% over 31 min and to 37% for 10 min, followed by a 3 min wash at 80% B and a 10 min re-equilibration at 4% B. Eluting peptides were converted to gas-phase ions by means of electrospray ionization and analysed on a Thermo Orbitrap Fusion (Thermo Scientific). Survey scans of peptide precursors from 375 to 1500 *m*/*z* were performed at 120 K resolution (at 200 *m*/*z*) with a target of 2×10^5^ ion counts. The maximum injection time was set to 150 ms. Tandem MS was performed by isolation at 1.2 Th using the quadrupole, higher-energy C-trap dissociation (HCD) fragmentation with normalized collision energy of 33, and rapid scan MS analysis in the ion trap. The MS2 ion count target was set to 5×10^3^. The maximum injection time was 200 ms. Precursors with charge state 2–6 were selected and sampled for MS2. The dynamic exclusion duration was set to 40 s with a 10 ppm tolerance around the selected precursor and its isotopes. Monoisotopic precursor selection was turned on. The instrument was run in top speed mode. The acquired tandem mass spectrometry (MS/MS) mass spectra were processed with SequestHT implemented on the Proteome Discoverer software version 2.2.0.388 for peptide and protein identifications against the *Homo sapiens* proteome (UP000005640, containing 71,544 proteins) and *Komagataella phaffii* proteome (UP000000314, containing 5073 proteins). The SequestHT node included the following parameters: precursor mass tolerance, 10 ppm and fragment mass tolerance, 0.6 Da. Dynamic modifications were: oxidation of M (+15.995 Da), acetyl of K (+42.011 Da), methyl of K (+14.016 Da), phospho of STY (+79.966 Da) and acetyl protein N-term (+42.011 Da). The static modification was carbamidomethyl of C (+57.021 Da). The level of confidence for peptide identifications was estimated with the Percolator node with decoy database search. The false discovery rate (FDR) was set at 0.01 based on the q-value.

### F-actin sedimentation assays and imaging

Actin (12.5 µM) in G-buffer was mixed with 2 mM MgCl_2_, 5 mM EGTA and 100 mM KCl for 1 h at 24°C. To make a 0.5 mM Latrunculin A (LatA) solution, 10 mM LatA in DMSO was first diluted to 2 mM with G-buffer, then further diluted to 0.5 mM in the reaction mixture (the final concentration of DMSO was 4%). The reaction mixture was ultracentrifuged in an Airfuge (Beckman Coulter) at 25 psi, room temperature for 30 min. Pellet and supernatant fractions were analysed on Coomassie-stained gels. For imaging actin filaments, actin (8.33 µM) in G-buffer was polymerized by addition of 2 mM MgCl_2_, 5 mM EGTA, 100 mM KCl and 2% polyethylene glycol 20,000 (#8.17018.1000, Merck Millipore) followed by incubation at 24°C for 1 h. 5% (v/v) Rhodamine–phalloidin (#R415, Thermo Fisher Scientific) or phalloidin–CF633 (#00046, Biotium) was added, and F-actin was imaged using an Andor Revolution XD spinning-disc confocal system on a Nikon Eclipse Ti inverted microscope, with a Nikon Plan Apo Lambda 100×1.45 NA oil immersion objective lens, a spinning-disc system (CSU-X1; Yokogawa) and an Andor iXon Ultra EMCCD camera. Images were acquired at the pixel size of 80 nm/pixel by using the Andor IQ3 software. Laser lines at a wavelength of 561 or 640 nm was used for excitation.

### Actin injection into zebrafish embryos

β- and γ-actin labelled with TMR (0.5 mg/ml) were diluted tenfold in low-ionic strength buffer [final composition: 5 mM HEPES (pH 7.4), 0.05 mM CaCl_2_, 0.0025% (w/v) NaN_3_, 0.2 mM ATP and 0.5 mM DTT] and polymerized actin was removed by ultracentrifugation with Airfuge (Beckman Coulter) at 25 psi for 10 min at 4°C.

Wild-type embryos were obtained by natural mating using standard procedures in accordance with institutional animal care regulations at the University of Warwick, UK. Approximately 0.113 nl of β- and γ-actin with TMR was injected into the blastoderm at the one-cell stage. Embryos were then dechorionated, embedded in 1% low-melting point agarose on no. 0 coverslips (MatTek 35 mm uncoated dishes), and imaged at the animal pole; while being maintained in ‘egg water’ at 26°C. Images were acquired by using Andor Revolution XD confocal systems, comprising a Nikon Eclipse Ti inverted microscope with a spinning-disc confocal Yokogawa CSU-X1 unit and an Andor iXon Ultra EMCCD camera. The β-actin-injected embryos were imaged using a Nikon Plan Apo Lambda 20×0.75 NA objective lens, with a final pixel size of 0.35 µm/pixel, via Andor IQ3 software. The γ-actin-injected embryos were imaged using a Nikon Plan Apo Lambda 10×0.45 NA objective lens, with a final pixel size of 0.8 µm/pixel, via Andor IQ3 software. A 561 nm laser line was used for excitation. All images were acquired with a *Z*-step size of 0.5 mm. The image stacks were projected along the *Z*-axis (maximum intensity) by using Fiji software (Schindelin et al., 2012).

### Cell culture

Immortalized (hTERT) diploid human retinal pigment epithelial (RPE1) cells were grown in DMEM/F-12 medium containing 10% fetal bovine serum (FBS), 2 mM L-glutamine, 100 U/ml penicillin and 100 μg/ml streptomycin. In each experiment, cells were plated, at 24 or 48 h before imaging, in a glass bottom FluoriDish (FD35-100, World Precision Instrument, Inc.).

### Actin injection into human RPE1 cells and imaging

β-actin labelled with Alexa Fluor 488 and γ-actin with TMR (0.25 or 0.5 mg/ml) were mixed in low-ionic strength buffer composed of 5 mM HEPES (pH 7.4), 0.05 mM CaCl_2_, 0.0025% (w/v) NaN_3_, 0.2 mM ATP and 0.5 mM DTT and polymerized actin was removed by ultracentrifugation with Airfuge (Beckman Coulter) at 25 psi for 10 min at 4°C.

Microinjection was performed under an Olympus IX71 microscope, equipped with a 40×0.6 NA long distance objective and the Integra TI micromanipulator (Research Instruments Ltd), by using the FemtoJet 4i microinjector (Eppendorf). G-actins were microinjected at room temperature into the cytoplasm of RPE1 cells by using pre-pulled Femtotips II needles (Eppendorf). Typically, in each experiment, cells from about ten different fields were injected. To visualise the DNA, RPE1 cells were incubated, 1 h before the injection, with DMEM/F-12 medium containing 0.5 µM SiR-DNA far-red probe (Spirochrome). Dynamic live movies and static live images were acquired after the microinjection was completed.

Live-cell imaging was performed on an Olympus DeltaVision Elite microscope (Applied Precision, LLC) equipped with a Photometrics CoolSNAP HQ camera (Roper Scientific) and a stage-top incubator (TokaiHit) to maintain cells at 37°C and 5% CO_2_. Temperature was further stabilized by using a microscope enclosure (Weather station; Precision Control) held at 37°C. Image stacks (ten 0.5 μm optical sections) were acquired using the softWoRX 5.5 software every 10 min for a 5 h period with a 40×1.3 NA oil-immersion objective. In each experiment, about ten individual fields (1024×1024 pixels) were imaged using the ‘point visit’ function in softWoRX 5.5. Original movies were then deconvolved with the softWoRX 5.5 software and processed with Fiji software (Schindelin et al., 2012).

Static live images were acquired using a confocal spinning-disc microscope (VOX UltraView; PerkinElmer, UK) equipped with a 100×1.4 NA oil-immersion objective and a Hamamatsu ORCA-R2 camera, controlled by Volocity 6.0 software (PerkinElmer) running on a Windows 7 64-bit (Microsoft, Redmond, WA) computer (IBM, New Castle, NY). The temperature on the spinning disc was stabilized by using a microscope enclosure (Solent Scientific) held at 37°C. Image stacks were acquired over 50 *Z*-slices separated by 0.2 µm for a single timepoint using the 488, 561 and 640 nm wavelength lasers. Spinning-disc images were exported from Volocity 6.0 in OME.TIFF format (The Open Microscopy Environment, UK). They were deconvolved in the 488 and 561 nm wavelengths within Huygens 4.1 software (SVI), using point spread functions (PSFs) calculated from 100 nm TetraSpeck fluorescent microspheres (Invitrogen) with the Huygens 4.1 PSF distiller. Images were deconvolved in the 640 nm wavelength within Huygens 4.1 using a theoretical PSF. Deconvolved images were exported from Huygens 4.1 in r3d format (Applied Precision, Slovakia) and processed with Fiji software (Schindelin et al., 2012).

## Supplementary Material

Supplementary information
