# Supplementary material for: Rapid production of pure recombinant actin isoforms in Pichia pastoris
Source: J Cell Sci. 2018 Apr 23;131(8):jcs213827. doi: 10.1242/jcs.213827 (PMC5976186; doi:10.1242/jcs.213827)
Supplement: Supplementary information [file joces-131-213827-s1.pdf]

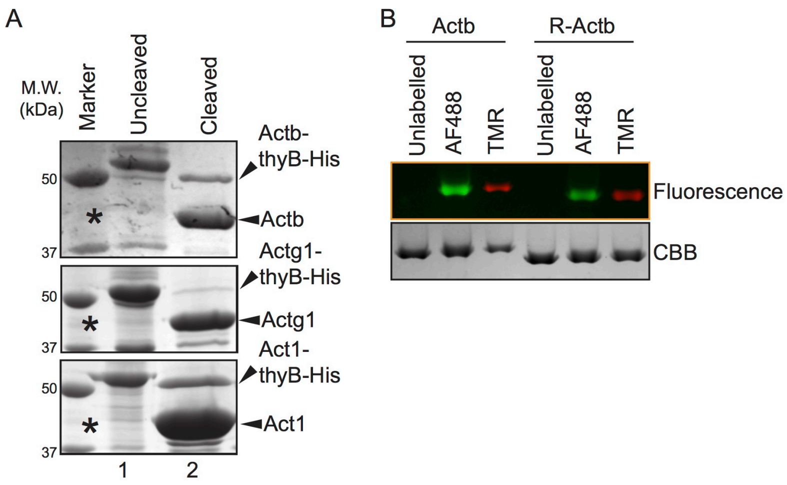

Supplemental figure 1. Purification of fission yeast Act1 and human non-muscle actins
(A) Endogenous P. pastoris actin does not co purify with the expressed recombinant actin. Proteins bound on nickel beads were eluted with SDS-PAGE sample buffer just before chymotrypsin cleavage and analysed by SDS-PAGE and CBB staining (Lane 1).
Eluates collected after chymotrypsin treatments were also loaded on SDS-PAGE gel (Lane 2). Note that a 43 kDa protein is visualized only after cleavage with chymotrypsin and not in the uncleaved sample, establishing that P. pastoris actin does not co-purify detectably.
(B) Fluorescent labelling of arginylated beta-actin with Alexa Fluor 488 C5 maleimide and TMR-5-maleimide.

Journal of Cell Science • Supplementary information

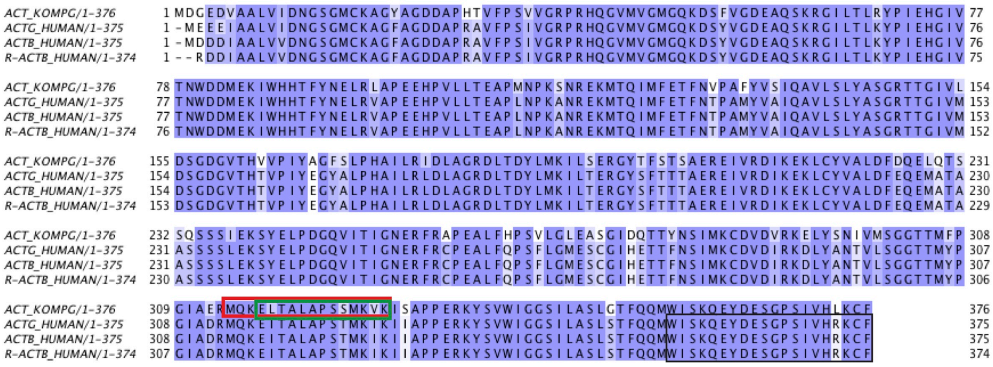

Supplemental figure 2. NanoLC-ESI-MS/MS analysis of actin proteins
Alignment of amino acid sequence of *P. pastoris* Act1 (ACT_KOMPG), β-actin (ACTB_HUMAN), γ-actin (ACTG_HUMAN), and arginylated β-actin (R_ACTB). Mass spectrometry identified tryptic peptides that covered the entire human β, γ, and R-β-actins. The only exception was the C-terminal CF residues, which were identified as part of a larger C-terminal peptide recovered upon cleavage the SDS-PAGE purified sample containing unfolded actin with chymotrypsin (black box).
The Pichia pastoris actin is predicted to contain ~ 6 unique peptides upon cleavage with trypsin that differentiate it from human β and γactins. We only found one area in all mass spectrometry experiments that may be consistent with arising from *P. pastoris* actin, marked with red and green boxes. In this area it was possible to assigned spectra to two miscleaved peptides with suggested methylation modifications (+14 Da). However, the same spectra could also be assigned to the human actins without additional methylation because there are two amino acid differences between human and *P. pastoris* sequences in this area, both with a molecular mass difference of 14 Da (serine to threonine and valine to isoleucine). It is possible that there may be a trace of *P. pastoris* methylated actin in the recombinant actin preparations. However, the simplest explanation, consistent with all observed spectra, is that un-methylated human actin (not *P. pastoris* actin) has been identified.

Journal of Cell Science • Supplementary information

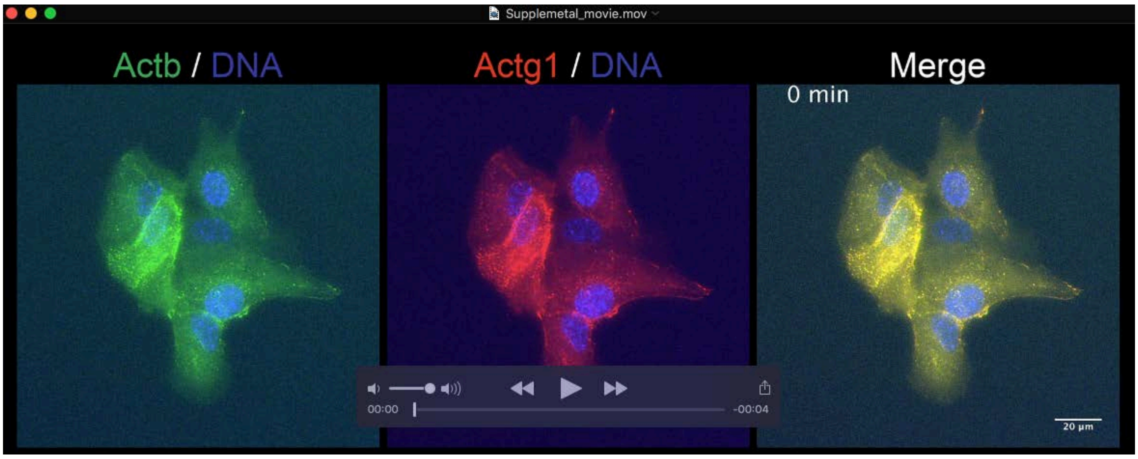

Supplemental movie. Time-lapse images of RPE cells injected with fluorescent labelled β- and γ-actins.

Time lapse images of microinjected β- and γ-actins in RPE cells. β- and γ-actin were labelled with Alexa Fluor 488 and TMR, respectively and injected into human RPE cells. Scale bar =20 µm. Images were acquired every 10 minutes.

Journal of Cell Science • Supplementary information

Supplemental table 1. Peptides from trypsin-digested samples detected by mass spectrometric analysis.

Click here to Download Table S1

Supplemental table 2. Peptides from chymotrypsin-digested samples detected by mass spectrometric analysis.

Click here to Download Table S2
